# The Analysis of Dynamic Changes and Prognosis of Posner–Schlossman Syndrome with Cytomegalovirus Infection and Antiviral Therapy

**DOI:** 10.1155/2021/6687929

**Published:** 2021-06-02

**Authors:** Qilian Sheng, Ruyi Zhai, Xintong Fan, Xiangmei Kong

**Affiliations:** ^1^Eye Institute and Department of Ophthalmology, Eye & ENT Hospital, Fudan University, Shanghai 200031, China; ^2^NHC Key Laboratory of Myopia (Fudan University), Key Laboratory of Myopia, Chinese Academy of Medical Sciences, Shanghai 200031, China; ^3^Shanghai Key Laboratory of Visual Impairment and Restoration, Shanghai 200031, China

## Abstract

**Purpose:**

To analyze how keratic precipitate (KP) morphology changes during Posner–Schlossman syndrome (PSS) prognosis and raise medication suggestions on 2% ganciclovir eye drops.

**Materials and Methods:**

Clinical retrospective cohort study in the Eye & ENT Hospital of Fudan University, Shanghai, China. The attacked eyes of 98 eligible subjects diagnosed unilateral PSS were enrolled between 2016 and 2019. All patients were treated with intraocular pressure-lowering drugs and anti-inflammatory steroids. 2% ganciclovir eye drops were given to cytomegalovirus (CMV) immunoglobulin G (IgG) correction ratio positive patients. Frequent follow-ups and examinations were performed. KP morphology was focused and categorized into coin-shaped, mutton-fat, and pigmented. Medical histories were noted. Multidimensional analysis was given.

**Results:**

Totally 47 patients in 98 achieved all-KP disappearance. Mean treatment time was (5.13 ± 3.66) weeks. Total KP disappearance was negatively correlated with mutton-fat and pigmented KPs at the first visit (*P*=0.020, *P*=0.007) and treatment time was also longer (*P*=0.018, *P*=0.014). Mean cumulative steroids dosage for 47 subjects was (159.66 ± 161.84) drops. CMV IgG correction ratio positive patients had smaller corneal endothelial cell density (*P* < 0.005) and larger cup-to-disc ratio (*P*=0.017) than negative subjects. Cumulative steroid treatment time was longer in the CMV-positive group, and overall dosage was also larger. However, due to 2% ganciclovir eye drops, daily steroid dosage was lower in the CMV-positive group.

**Conclusions:**

The disappearance of mutton-fat and pigmented KPs needed longer treatment time. Paired aqueous humor and serum CMV IgG tests were recommended in PSS patients with coin-shaped KPs. 2% ganciclovir eye drops improved prognosis; and steroids dosage reduced significantly.

## 1. Introduction

Posner–Schlossman syndrome (PSS) is a special form of anterior uveitis with glaucoma mainly seen in young adults between 20 and 50 years old, characterized by nongranulomatous uveitis with marked increase in intraocular pressure (IOP) [1, 2]. During the acute attack phase, short duration of ocular discomfort and blurred vision may be present [3]. A key feature of PSS is the sharp IOP rise accompanied by anterior chamber active inflammation. On clinical examination, mild ciliary flush, corneal edema, iris changes, and a few gray-white medium-to-large granulomatous keratic precipitates (KPs) on the central or peripheral cornea are present with minimal flare [1–4]. Composed of epithelioid cells, lymphocytes, and polymorphonuclear cells, KPs are aggregates of inflammatory cells on the corneal endothelium [5]. According to the view under a slit-lamp biomicroscope, KPs can be classified into heterogeneous morphologic categories, including mutton-fat KPs, stellate KPs, and coin-shaped KPs [5–7]. During the whole disease course, KP morphology and quantity may change over time [6]. Being an intuitive index under a slit-lamp biomicroscope, dynamic KP morphology may reflect PSS progression and medication efficacy. Concise classifications of KP under slit-lamp are observed and discussed in this study.

The pathogenesis of PSS has not been confirmed yet, but recent polymerase chain reaction (PCR) techniques have shown that cytomegalovirus (CMV), a member of the Herpesviridae family, could be responsible for PSS cases previously considered idiopathic [8–12]. In Chinese population, aqueous humor CMV antibody-positive patients accounted for 60% in our preliminary results [13]. Aqueous humor and serum CMV antibody examination and antivirus therapy have not been a routine in clinical practice. Several case reports have found CMV examinations effective, but intravenous and oral ganciclovir therapy had limitations in application or side effects [13–16]. Topical ganciclovir (mainly 0.15% gel) has received better response [16, 17]. A larger retrospective trial of topical 2% solution is tested in our study.

CMV infection, PSS prognosis, and KP morphology changes reflect pathogenesis, progression, and manifestation, respectively. Deepening the current understanding of PSS and raising effective medication suggestions are of high value in clinical practice.

## 2. Materials and Methods

### 2.1. The Subjects

Ninety-eight consecutive immunocompetent patients diagnosed unilateral PSS between 2016 and 2019 in the Eye & ENT Hospital of Fudan University, Shanghai, were included in this study. The study was approved by the Ethic Committee of Eye & ENT Hospital and was conducted in accordance with the Declaration of Helsinki (1964). Informed consent was obtained from each patient.

Patients had to be older than 10 years and younger than 80 years. Patients must be clearly diagnosed PSS, which was classified as follows: (1) recurrent attacks of mild, unilateral, nongranulomatous anterior uveitis accompanied by markedly elevated IOP, small white KPs on the endothelial surface of the central cornea, open angle, no posterior synechia, and no inflammatory lesions in the posterior segment of the eye; and (2) the IOP and anterior chamber inflammation returned normal between attacks. Patients had to be in the attack phase of PSS, and a complete ocular examination of this condition was performed as the baseline information.

The exclusion criteria were as follows: (1) primary glaucoma or elevated IOP caused by other known factors; (2) previous trauma, uveitis caused by herpes simplex virus or herpes zoster virus, retinal or corneal diseases; (3) allergic to any steroids, IOP-lowering drugs, or ganciclovir; (4) liver or kidney dysfunction; and (5) pregnant or lactating women.

Demographics and medical history were obtained. IOP was measured using a Goldmann applanation tonometer. KP morphology was observed using a slit-lamp biomicroscope.

All patients were treated with topical steroids (mainly 1% prednisolone (Allergan Pharmaceuticals, Ireland), 0.1% dexamethasone (ALCON-COUVREUR, Belgium), or 0.1% fluorometholone (Santen Pharmaceuticals, Japan)) and/or IOP-lowering medications as necessary after the first visit (mainly beta-blockers, alpha-2 adrenergic agonists, or carbonic anhydrase inhibitor). Dosage and frequency were adjusted based on each subject's condition. Additional 2% ganciclovir solution was provided for CMV-positive patients. Once the diagnosis was confirmed, patients were treated with 2% ganciclovir at least 3 times daily (mean: 4 times/day), tapered based on anterior chamber inflammation over the course of 3 months, and subsequently kept on long-term maintenance therapy (3–4 times/day). Steroids tapered off 1 time daily every week and finally stopped based on IOP and anterior chamber clinical response. Patients were followed up on a mean basis of three-month interval for up to 3 years. During routine follow-up, IOP, KP morphology, dosage, and response to current medication were regularly recorded. The cup-to-disc ratio (CDR) and corneal endothelial cell density (ECD) were measured as safety indicators. Images were captured from central cornea and analyzed using IMAGEnet 2000 software (TOPCON).

### 2.2. Aqueous Humor and Antibody Analysis

The anterior chambers were tapped when patients presented with a new episode of hypertension uveitis, prior to starting new medication. All the patients underwent anterior chamber paracentesis only once. After obtaining the informed consent, anterior chamber paracentesis was performed using an aseptic technique under a slit lamp. Paired aqueous humor and serum samples were assayed to detect CMV immunoglobulin G (IgG) by enzyme-linked immunosorbent assay (ELISA; Virion\Serion, Germany) as described by Wang et al. [18] Other possible pathogens were also detected, including herpes virus 1 and 2, varicella zoster virus, rubella virus, and toxoplasma gondii. The total amount of IgG in the aqueous humor and serum was determined by a radial immunodiffusion technique. When CMV IgG was detected in the aqueous humor and the correction ratio of aqueous humor/serum was greater than 0.20, CMV positive was considered [18, 19]. Otherwise, CMV negative was defined.

### 2.3. Preparation of 2% Ganciclovir Eye Drops

0.25 g dry powder of ganciclovir for injection (Luoxin, China) was dissolved in 2.5 mL water for injection (GIBCO WFI; Thermo Fisher Scientific, USA); and 1.25 mL ganciclovir solution was mixed with 5 mL 0.3% sodium hyaluronate eye drops (Santen, Japan). By this method, 125 mg : 6.25 mL (2%) ganciclovir eye drops are made. The 2% ganciclovir eye drops were prepared by the Pharmacy of Eye & ENT Hospital (qualified for preparing clinical solutions) and approved by the Ethics Committee of Eye & ENT Hospital of Fudan University.

### 2.4. Classification of Patterns of KP and Related Definitions

KP morphology was classified into three patterns: coin-shaped KP, mutton-fat KP, and pigmented KP.

End point was reached if all KPs in the affected eye disappeared, and no side effect was detected on both sides. The treatment time required from the first onset to the end point was calculated as mean ± standard deviation (SD). Patients suffering from side effects, being lost follow-up, or exiting study due to any reason were not included in the final analysis.

Cases exempted from steroids referred to those who could maintain the current IOP level of both sides (within 21 mmHg), and no inflammatory manifestations recurred in the anterior chamber (including fresh KPs, Tyndall reaction, and anterior chamber cells) without relying on steroids. If IOP elevated or inflammation recurred once current steroid dosage reduced or withdrew, these cases were defined steroid-dependent.

### 2.5. Statistical Analysis

Statistical analysis was performed with SPSS, version 21.0, software. *χ*^2^ tests were used to compare the three KP patterns of all patients in the attack phase and at the end point. Student's *t*-tests were used to compare indicators in CMV-positive and CMV-negative groups. A *P* value of less than 0.05 was considered significant. Logistic regression, Cox survival analysis, and multiple linear regression were used to compare the impact of three KP patterns on treatment outcome and time. All analyses were carried out on the basis of the protocol definitions discussed above. No subject was excluded.

## 3. Results and Discussion

### 3.1. Subjects and Clinical Manifestations

Totally 98 affected eyes of 98 subjects confirmed eligible were included in the study. The demographics of patients and ocular examination results at first visit are shown in Table 1.

**Figure 1:**
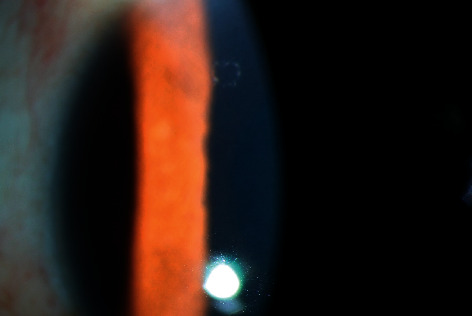
Coin-shaped KP under a slit lamp. Color photograph of the left eye of a 40-year-old female patient with a 2-year PSS course. A typical coin-shaped KP can be seen in the upper half of the photograph and disappeared after 2% ganciclovir eye drops application.

**Figure 2:**
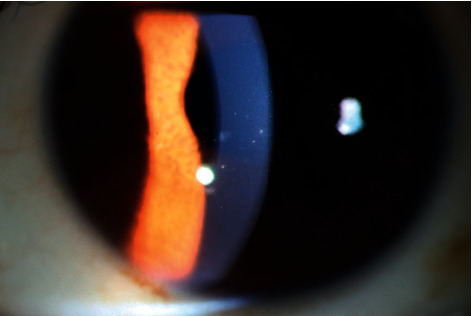
Mutton-fat KP under a slit lamp. Color photograph of the left eye of a 40-year-old male patient shows typical mutton-fat KP under a slit lamp. Several mutton-fat KPs can be seen in the middle and lower position.

**Figure 3:**
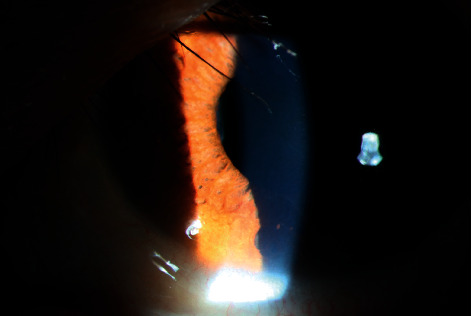
Pigmented KP under a slit lamp. Color photograph of the left eye of a 46-year-old female patient shows typical pigmented KP and moth-eaten iris depigmentation under a slit lamp. Two pigmented KPs can be seen in the lower position. PSS = Posner–Schlossman syndrome; KP = keratic precipitate.

Among all the patients, men were more susceptible than women (62.24%). IOP and coin-shaped and mutton-fat KPs all declined significantly during follow-up (*P* < 0.001) (Figures 1 and 2). Pigmented KPs remained in a similarly low proportion (13% to 11%, *P*=0.66) (Figure 3).

### 3.2. Relationship between KP Morphology, CMV Infection, and PSS Prognosis

Totally 47 patients in 98 achieved all-KP disappearance within a maximum of 3-year follow-up period (47.96%). Mean treatment time was (5.13 ± 3.66) weeks. Through Cox survival analysis, the presence of mutton-fat and pigmented KPs was negatively correlated with final KP disappearance (*P*=0.020, *P*=0.007). Through multilinear regression, the greater the possibility of the presence of mutton-fat (*P*=0.018) and pigmented KPs (*P*=0.014) at the first visit, the longer the treatment time. Coin-shaped KP was found significant in neither judging treatment outcome nor time (*P*=0.436, *P*=0.699).

Mean cumulative steroids dosage for 47 subjects was (159.66 ± 161.84) drops. Particularly, pigmented KP was positively correlated with cumulative steroids dosage (*P*=0.005).

Among the 98 cases, 64 had longer steroids history and larger daily dosage compared with other 34 cases (2.48 ± 0.91 drops compared with 1.82 ± 1.24 drops daily, *P*=0.003). Their IOP was also higher (24.83 ± 11.02 mmHg compared with 19.14 ± 8.21 mmHg, *P*=0.009). These patients underwent paired aqueous humor and serum CMV IgG tests and aqueous humor pathogen tests. None was found positive for herpes virus 1 or 2, varicella zoster virus, rubella virus, or toxoplasma gondii. Thirty-three were CMV-positive and 28 were negative. The number of coin-shaped, mutton-fat, and pigmented KPs showed no significant difference in comparison of CMV IgG correction ratio positive (*N* = 36) and negative (*N* = 28) groups. The ECD of CMV-positive patients was significantly smaller than that of negative ones (*P* < 0.005), and CDR was larger (*P*=0.017). Thirty in 64 achieved total KP disappearance, including 18 from the CMV-positive group and 12 from the CMV-negative group. Seventeen in 30 were exempted from steroids at the end point, composed of 10 from the CMV-positive and 7 from the CMV-negative group. In general, these 17 cases had better visual acuity (VA) (0.15, measured in logMAR) and lower IOP (23.71 mmHg) at the first visit, compared with other 13 steroid-dependent cases (0.24 and 28.31 mmHg, respectively) (Table 2).

No significant side effects, other than ocular discomfort from topical ganciclovir application or slight ocular surface toxicity, were noted in all the patients.

Current IOP-lowering and anti-inflammation treatment are not sufficient in controlling PSS progression, especially in CMV-positive patients, which accounted for nearly 60% according to our previous studies [13]. The application criteria for ganciclovir had not been clarified; effective formulation also needs to be tested and promoted. Total KP disappearance under a slit lamp positively reflects the subsiding of anterior chamber inflammation and better prognosis.

We classified KPs into three types mostly observed in PSS under a slit lamp [20, 21]. We found different KP patterns changed differently during PSS prognosis, and disappearance of specific KPs was correlated with the treatment period. Anterior chamber CMV infection posed threat to the corneal endothelium.

Resulted from immune reaction [20], high percent of mutton-fat KP presence indicates severe fresh attack. Pigmented KP is more a sign of historical attack, which is harder to eliminate completely. However, continuous steroids still worked in eliminating all patterns of KPs.

Chan et al. explained that coin-shaped KPs had a positive predictive value of 90.9% for CMV infection [2]. However, the presence rate of coin-shaped KPs was similar in CMV-positive and CMV-negative groups in our study, and through analysis, we did not find exact correlation between coin-shaped KP and treatment outcome. This seemed in conflict with previous observations, but actually some patients had medication before coming to our hospital. They were in the attack phase of PSS progression rather than the initial stage. This may interfere with clinical manifestations and was difficult to avoid. Besides, it was inaccurate to conclude the CMV-negative group must have a low proportion of coin-shaped KPs. However, the rapid decline of coin-shaped KPs suggested our topical antivirus therapy was efficient, which was consistent with Su's findings that prepared 2% ganciclovir eye drops reduced CMV viral load [14]. Endotheliitis caused by CMV infection produces detrimental effect on the corneal endothelium [22, 23]. Our study confirmed the damage which led to thinner endothelial thickness and larger CDR in the CMV-positive group. Paired aqueous humor and serum CMV IgG tests are highly recommended in PSS patients with coin-shaped KPs. Though CMV IgM can be supplemental evidence for CMV infection, it was difficult for IgM to cross the blood-brain barrier, which caused a rather low detection rate in aqueous humor. CMV-positive patients consumed more steroids and took longer time to eliminate all KPs. This also supports that CMV infection worsens PSS progression and is more difficult to control. However, at the end point, daily steroid dosage for the CMV-positive group was lower. This may be benefited from ganciclovir application. The conditions of cases exempted from steroids were similar in the two groups in clinical examinations, including anterior chamber manifestations, CDR and VA, which indicated our ganciclovir medication, helped CMV-positive patients achieve similar prognosis to CMV-negative patients. After all, there have not been controlled studies comparing PSS prognosis with topical 2% ganciclovir eye drops. A limited number of studies [13, 15–17, 24] had reported the efficacy of topical application of ganciclovir. It could penetrate the corneal stroma and achieve therapeutic levels in the aqueous humor. Current 0.15% ganciclovir gel showed better efficacy, but the frequent application was not convenient enough. According to the results of a comparative study by Su et al., the 2% ganciclovir solution effectively cleared CMV viral load and controlled IOP in CMV-positive PSS patients. Having been tested for several years, our 2% ganciclovir eye drops received promising results [13]. The high concentration of ganciclovir quickly controlled uveitis attack and protected the endothelium from being damaged by long-lasting active CMV. After a mean treatment period of (5.40 ± 2.70) weeks, all the three KP patterns reduced. IOP and steroid dependence also declined in 50 cases. These preliminary small-size results served our current study. With expectation to stronger clinical evidence, a larger multicenter controlled study is still needed in future practice.

Comparing steroid-dependent cases, we found cases exempted from steroids had better VA and lower IOP. Although the subject size did not support *t*-test, the huge differences of the means seemed meaningful. Although reached all-KP disappearance, patients still need steroids to maintain current visual function.

Our study had several limitations. First, total KP disappearance is not enough to fully represent that PSS has already been under control. However, it is a credible indicator of stable anterior chamber and has potential value in clinical practice. Second, we did not introduce aqueous humor CMV DNA PCR, so it is difficult to match KP number with CMV copies. A quantitative correlation is still needed in further studies. Meanwhile, the sample size needs to be expanded to perform *t* tests when observing case characteristics exempted from steroids.

## 4. Conclusions

Overall, KP morphology changed differently during PSS prognosis and may have a predictive value in estimating and comparing medication time. Paired aqueous humor and serum CMV IgG tests are highly recommended in patients with coin-shaped KPs. 2% ganciclovir eye drops have positive efficacy in CMV-positive PSS patients and may shed light on future pathogenetic treatment.

## Figures and Tables

**Table 1:** Baseline characteristics of the 98 patients with Posner–Schlossman syndrome.

Characteristics	Value	*P* value
Gender (male/female)	61/37	
Eye (OD/OS)	52/46	
Age, years	40.04 ± 13.15 (18–69, median 38.5)	
Age of first attack	34.66 ± 12.04 (median, 32.8)	
Disease course, years	6.40 ± 6.19	
IOP in the attack phase, mmHg	22.85 ± 10.45	
IOP at the last visit, mmHg	15.89 ± 4.59	**<0.001**
*Coin-shaped KP*		
In the attack phase	25.51% (25/98)	**<0.001** ^*∗*^
At the last visit	3.06% (3/98)	

*Mutton-fat KP*		
In the attack phase	77.55% (76/98)	**<0.001** ^*∗*^
At the last visit	43.88% (42/98)	

*Pigmented KP*		
In the attack phase	13.27% (13/98)	0.66^*∗*^
At the last visit	11.22% (11/98)	

Values are mean ± standard deviation (range). OD = oculus dexter, OS = oculus seminar, IOP = intraocular pressure, KP = keratic precipitate. ^*∗*^*χ*^2^ test.

**Table 2:** Comparison of patients from the CMV-positive and CMV-negative group.

	CMV positive (*N* = 36)	CMV negative (*N* = 28)	*P* value
IOP of the affected eye, mmHg	24.25 ± 11.17	25.57 ± 10.98	—
Coin-shaped KP			
At the onset	9/36	8/28	—
At the last visit	1/36	1/28	—
ECD, cells/mm^2^	2278.45 ± 355.63	2557.62 ± 314.27	0.005^*∗*^
CDR	0.58 ± 0.22	0.46 ± 0.16	0.017^*∗*^
Number of cases achieved all-KP disappearance	18/36	12/28	—
Cumulative steroids dosage, drops	204.56 ± 195.67	129.50 ± 125.02	—
Cumulative ganciclovir eye drops dosage (drops)	567.00 ± 867.45	0	—
Cumulative treatment time (weeks)	21.44 ± 34.67	10.08 ± 7.53	—
Steroids dosage per day at the last visit (times/day)	0.61 ± 0.78	0.75 ± 0.97	—
Number of cases exempted from steroids	10/18	7/12	—

IOP = intraocular pressure. CMV = cytomegalovirus. ECD = endothelial cell density. CDR = cup-to-disc ratio. KP = keratic precipitate. ^*∗*^Student's *t*-tests.

## Data Availability

The patients' data used to support the findings of this study are available from the corresponding author upon request.

## References

[1] Megaw R., Agarwal P. K. (2017). Posner-Schlossman syndrome. *Survey of Ophthalmology*.

[2] Chan N. S.-W., Chee S.-P., Caspers L., Bodaghi B. (2017). Clinical features of CMV-associated anterior uveitis. *Ocular Immunology and Inflammation*.

[3] Shazly T. A., Aljajeh M., Latina M. A. (2011). Posner-Schlossman glaucomatocyclitic crisis. *Semin Ophthalmol*.

[4] Jap A., Sivakumar M., Chee S.-P. (2001). Is Posner Schlossman syndrome benign?. *Ophthalmology*.

[5] Mocan M. C., Kadayifcilar S., Irkec M. (2009). Keratic precipitate morphology in uveitic syndromes including Behçet’s disease as evaluated with in vivo confocal microscopy. *Eye*.

[6] Kanavi M. R., Soheilian M. (2011). Confocal scan features of keratic precipitates in granulomatous versus nongranulomatous uveitis. *Journal of Ophthalmic & Vision Research*.

[7] Pillai C. T., Dua H. S., Azuara-Blanco A., Sarhan A. R. (2000). Evaluation of corneal endothelium and keratic precipitates by specular microscopy in anterior uveitis. *British Journal of Ophthalmology*.

[8] Takusagawa H. L., Liu Y., Wiggs J. L. (2011). Infectious theories of Posner-Schlossman syndrome. *International Ophthalmology Clinics*.

[9] Joye A., Gonzales J. A. (2018). Ocular manifestations of cytomegalovirus in immunocompetent hosts. *Current Opinion in Ophthalmology*.

[10] Chan N. S. W., Chee S. P. (2019). Demystifying viral anterior uveitis: a review. *Clinical & Experimental Ophthalmology*.

[11] Rodier-Bonifas C., Cornut P.-L., Billaud G., Lina B., Burillon C., Denis P. (2011). Cytomegalovirus research using polymerase chain reaction in Posner-Schlossman syndrome. *Journal Français d’Ophtalmologie*.

[12] Martín Ramírez A., Cardeñoso Domingo L., González Guijarro J. J. (2019). PCR multiplex for CMV detection in patients with anterior uveitis. *Ocular Immunology and Inflammation*.

[13] Zhai R. Y., Xu H., Kong X. M. (2018). Effect of 2% ganciclovir eye drops on cytomegalovirus positive Posner-Schlossman syndrome. *Zhonghua Yan Ke Za Zhi*.

[14] Su C.-C., Hu F.-R., Wang T.-H. (2014). Clinical outcomes in cytomegalovirus-positive Posner-Schlossman syndrome patients treated with topical ganciclovir therapy. *American Journal of Ophthalmology*.

[15] Sobolewska B., Deuter C., Doycheva D., Zierhut M. (2014). Long-term oral therapy with valganciclovir in patients with Posner-Schlossman syndrome. *Graefe’s Archive for Clinical and Experimental Ophthalmology*.

[16] Antoun J., Willermain F., Makhoul D., Motulsky E., Caspers L., Relvas L. J. (2017). Topical ganciclovir in cytomegalovirus anterior uveitis. *Journal of Ocular Pharmacology and Therapeutics*.

[17] Wong J. X. H., Agrawal R., Wong E. P. Y., Teoh S. C. (2016). Efficacy and safety of topical ganciclovir in the management of cytomegalovirus (CMV)-related anterior uveitis. *Journal of Ophthalmic Inflammation and Infection*.

[18] Wang X. L., Wang Z. J., Tang L., Cao W. W., Sun X. H. (2017). Determination of cytomegalovirus IgG synthesis by the albumin correction in the aqueous humor of Posner-Schlossman syndrome. *Zhonghua Yan Ke Za Zhi*.

[19] Dernouchamps J. P., Magnusson C. G. M., Michiels J., Masson P. L. (1985). Immunoglobulin E in aqueous humour. *Experimental Eye Research*.

[20] Yoshimura A., Araki-Sasaki K., Toyokawa N., Fujiwara R., Jho N., Gomi F. (2020). Relationships between the clinical characteristics and copy numbers of DNA of cytomegalovirus determined by real-time PCR. *International Ophthalmology*.

[21] Moshirfar M., Murri M. S., Shah T. J. (2019). A review of corneal endotheliitis and endotheliopathy: differential diagnosis, evaluation, and treatment. *Ophthalmology and Therapy*.

[22] Koizumi N., Inatomi T., Suzuki T. (2015). Clinical features and management of cytomegalovirus corneal endotheliitis: analysis of 106 cases from the Japan corneal endotheliitis study. *British Journal of Ophthalmology*.

[23] Ang M., Sng C. C. A., Chee S.-P., Tan D. T. H., Mehta J. S. (2013). Outcomes of corneal transplantation for irreversible corneal decompensation secondary to corneal endotheliitis in Asian eyes. *American Journal of Ophthalmology*.

[24] Koizumi N., Miyazaki D., Inoue T. (2017). The effect of topical application of 0.15% ganciclovir gel on cytomegalovirus corneal endotheliitis. *The British Journal of Ophthalmology*.

